# FLAIR* to visualize veins in white matter lesions: A new tool for the diagnosis of multiple sclerosis?

**DOI:** 10.1007/s00330-017-4822-z

**Published:** 2017-04-13

**Authors:** T. Campion, R. J. P. Smith, D. R. Altmann, G. C. Brito, B. P. Turner, J. Evanson, I. C. George, P. Sati, D. S. Reich, M. E. Miquel, K. Schmierer

**Affiliations:** 1 Blizard Institute (Neuroscience), Queen Mary University of London, London, UK; 2 Barts Health NHS Trust, Emergency Care and Acute Medicine Clinical Academic Group Neuroscience, The Royal London Hospital, Whitechapel Road, London, UK; 3 Gloucestershire Hospitals NHS Trust, Cheltenham, UK; 4 Department of Medical Statistics, London School of Hygiene and Tropical Medicine, London, UK; 5 Translational Neuroradiology Section, National Institute of Neurological Disorders and Stroke, NIH, Bethesda, MD USA; 6 Department of Neurology, Yale School of Medicine, New Haven, CT USA; 7 William Harvey Research Institute (Cardiovascular Biomedical Research Unit), Queen Mary University of London, London, UK

**Keywords:** Multiple sclerosis, MRI, White matter, Neuroimaging, Central vein

## Abstract

***Objective*:**

To explore the potential of a post-processing technique combining FLAIR and T_2_* (FLAIR*) to distinguish between lesions caused by multiple sclerosis (MS) from cerebral small vessel disease (SVD) in a clinical setting.

**Methods:**

FLAIR and T_2_* head datasets acquired at 3T of 25 people with relapsing MS (pwRMS) and ten with pwSVD were used. After post-processing, FLAIR* maps were used to determine the proportion of white matter lesions (WML) showing the ‘vein in lesion’ sign (VIL), a characteristic histopathological feature of MS plaques. Sensitivity and specificity of MS diagnosis were examined on the basis of >45% VIL^+^ and >60% VIL^+^ WML, and compared with current dissemination in space (DIS) MRI criteria.

**Results:**

All pwRMS had >45% VIL^+^ WML (range 58–100%) whilst in pwSVD the proportion of VIL^+^ WML was significantly lower (0–64%; mean 32±20%). Sensitivity based on >45% VIL^+^ was 100% and specificity 80% whilst with >60% VIL^+^ as the criterion, sensitivity was 96% and specificity 90%. DIS criteria had 96% sensitivity and 40% specificity.

**Conclusion:**

FLAIR* enables VIL^+^ WML detection in a clinical setting, facilitating differentiation of MS from SVD based on brain MRI.

***Key points*:**

• FLAIR* in a clinical setting allows visualization of veins in white matter lesions.

*• Significant proportions of MS lesions demonstrate a vein in lesion on MRI.*

*• Microangiopathic lesions demonstrate a lower proportion of intralesional veins than MS lesions.*

*• Intralesional vein-based criteria may complement current MRI criteria for MS diagnosis.*

## Introduction

No noninvasive test result is fully specific for the diagnosis of multiple sclerosis (MS), one of the most common conditions causing chronic neurological disability. The current diagnostic criteria for MS (‘McDonald’ criteria) are based on clinical and paraclinical evidence, including magnetic resonance imaging (MRI), of dissemination in time (DIT) and space (DIS) of lesions suggestive of inflammatory demyelination. These criteria also rely on the exclusion of alternative conditions that would better explain a patient’s symptoms, signs and results of investigations [1]. The stipulation of ‘no better explanation’ underpins the character of MS as a diagnosis of exclusion.

MRI of the brain and spinal cord is the single most important investigation in the diagnostic work-up of people suspected of having MS, and serves both the diagnostic criteria laid down by the International Panel [1] and the exclusion of differential diagnoses. However, lesions suggestive of demyelination detected on conventional T_2_-weighted MRI may in fact have a different pathological substrate, such as cerebral small vessel disease (SVD), migraine or infections [2]. The probability of one of these alternative diagnoses may depend, for example, on age, vascular risk factors or genetic background. Strict adherence to the current criteria can therefore delay the definitive diagnosis of MS and, as a result, disease-modifying treatment (DMT). Given the evidence that treatment of people with MS, particularly those with a relapsing course (pwRMS), is most effective when started early, such delay may be clinically important [3–5].

Another limitation of current MRI techniques is apparent in people with MS who also have risk factors for SVD. Co-morbidity may compromise correct allocation of new lesions to their cause [6], and such uncertainty may directly impact on treatment decisions [7, 8].

In order to further improve MRI as a tool to support a diagnosis of MS, one of its characteristic histological features has recently been revisited: the vein about which MS lesions almost invariably evolve [9, 10]. This perivenous morphology of MS lesions appears to have become accessible *in vivo* using MRI techniques susceptible to iron in deoxygenated haemoglobin, such as susceptibility or T_2_*-weighted imaging [11].

In a study using T_2_*-weighted imaging at 7T, the detection of ‘veins in lesions’ (VIL) suggested VIL may be useful as a diagnostic marker for MS [12]. A further study by the same group suggested a proportion of 40% or more VIL positive (VIL^+^) white matter lesions (WML) distinguished people with MS (pwMS) from people with WML of a different aetiology with 100% positive and negative predictive values [13].

Whilst 7T MRI currently provides the best platform in terms of signal-to-noise and resolution to detect WML and cerebral veins alike, 7T scanners are not widely available, particularly in clinical settings, and a substantial number of WML and cerebral veins can also be visualized at 3T [14]. At 3T, a proportion of 45% VIL^+^ WML has been shown to correctly categorize patients as having MS or SVD lesions [15].

In the study reported here, T_2_*-weighted 3D echo-planar-imaging (3D EPI), to detect VIL, and T_2_-weighted fluid attenuated inversion recovery (FLAIR) sequences, to detect WML, were acquired at 3T and combined to generate FLAIR* images, first described by Sati and co-workers [16].

Using datasets acquired in a routine clinical setting we explored the sensitivity and specificity of a set of criteria based on FLAIR* for comparison with the revised International Panel MRI criteria currently used to support a diagnosis of MS. We further compared our results to a group of patients with WML and a clinical profile consistent with SVD to determine whether the proportion of VIL^+^ WML could be useful in differentiating MS from SVD.

## Materials and methods

### Subjects

This study was approved by the National Research Ethics Committee North West – Haydock (15/NW/0065) and undertaken at a single centre, The Royal London Hospital (RLH), Barts Health NHS Trust, London, UK. Datasets were used of patients who had undergone MRI as part of routine care. Written informed consent was obtained prior to any study procedure. Principal sources of referrals were the Neurology and Neuroinflammation services of the RLH, with additional referrals facilitated by the National Institute of Health Research (NIHR), North Thames Clinical Research Network.

For inclusion in the study MRI brain studies had to show at least one white matter lesion. Datasets of pwRMS were included if the diagnosis was confirmed according to the most recent International Panel criteria [1]. Datasets of people with SVD (pwSVD) were included if they (i) did not have a clinical diagnosis of MS (and were not suspected of having MS) and (ii) had at least two of six risk factors for SVD (diabetes, high blood pressure, smoking, hypercholesterolaemia, ischaemic heart disease, peripheral vascular disease [17]). Datasets of patients with any additional CNS pathology were excluded.

### Data acquisition

All images were acquired on a Philips Achieva 3T TX system (Philips Healthcare, Best, The Netherlands) based at St Barts Hospital of Barts Health NHS Trust using either a 16-element neurovascular coil or an 8-element head coil. The protocol included a T_2_*-weighted 3D segmented EPI sequence (TE 29 ms, TR 53 ms, flip angle 10°, EPI factor 15, field of view 240×240×180 mm^3^, 0.55×0.55×0.55 mm^3^ resolution, SENSE acceleration 2×2, total acquisition time 3 min 50 s) [18] and a 3D FLAIR sequence (VISTA protocol, TE 372 ms, TR 4800 ms, TI 1600 ms, field of view 240×240×180 mm^3^, 1×1×1 mm^3^ resolution, SENSE acceleration 2×2.6, total acquisition time 6 min) after injection of 10 ml of 0.5 mmol/ml gadoteric acid contrast agent. Contrast was injected manually right before the T_2_* sequence was acquired. FLAIR was acquired 13 min post contrast administration. The scanner manufacturer provided both sequences. 3D T_1_ (fast field echo; before and after administration of gadolinium) and 3D T_2_ (turbo spin echo) sequences were also obtained in order to assess lesions according to the McDonald criteria. Due to our local scanning protocols, these scanning parameters differ slightly from those described by Sati et al. [16]; a standard contrast dose with manual injection was used instead of a weight-adjusted contrast dose via power injector, and thus the delay to the FLAIR sequence was longer.

### Image processing

**Fig. 1:**
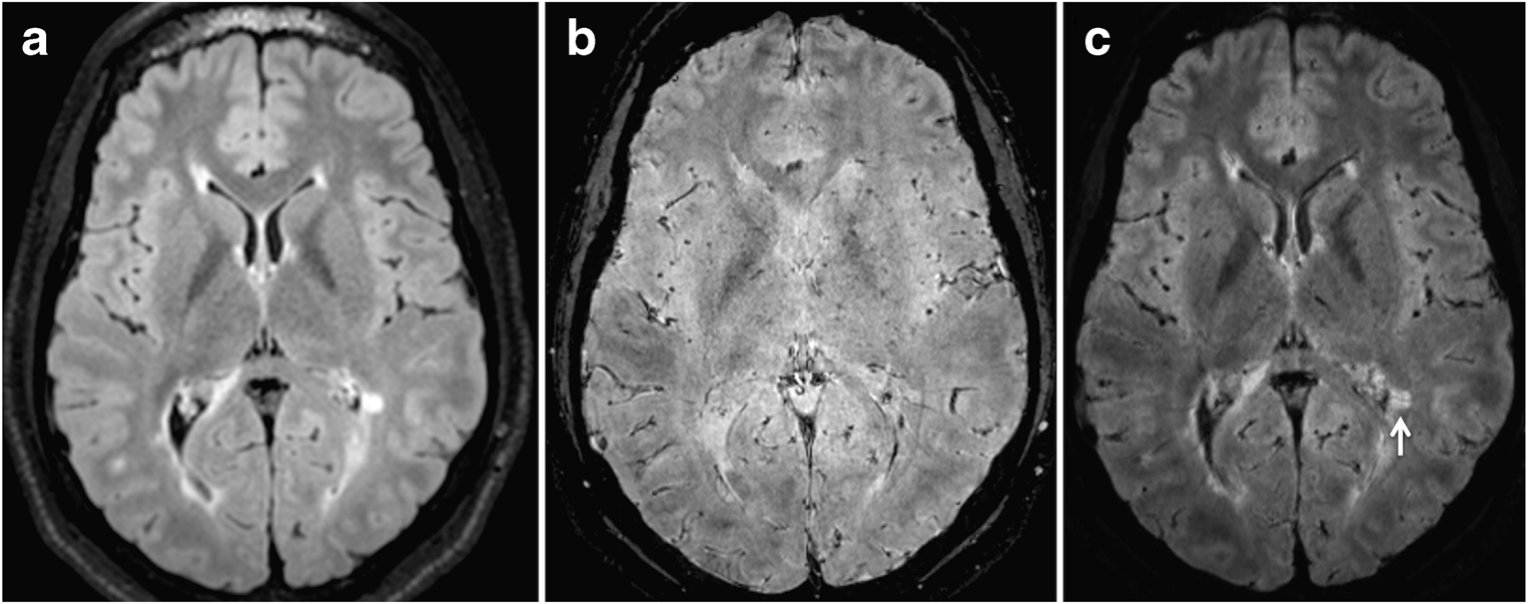
Construction of FLAIR* images. (**a**) FLAIR, axial slice. (**b**) T_2_*, axial slice. (**c**) Combined images create FLAIR*, axial slice. White arrow indicates intralesional vein

FLAIR* images were constructed using the FLAIR and T_2_* datasets using MIPAV (mipav.cit.nih.gov) and JIST (www.nitrc.org/projects/jist/) image processing software. Post-processing was conducted using a processing pipeline as described previously [16]. All images were first reformatted to the axial orientation without interpolation. To correct for motion between acquisitions, the FLAIR dataset was co-registered to the 3D EPI sequence using a rigid registration with six degrees of freedom, normalised mutual information as the cost function, and windowed sinc interpolation. The registered FLAIR images were then interpolated to the same spatial resolution and multiplied to the 3D EPI sequence to obtain the FLAIR* images (Fig. 1).

### Data analysis

**Fig. 2:**
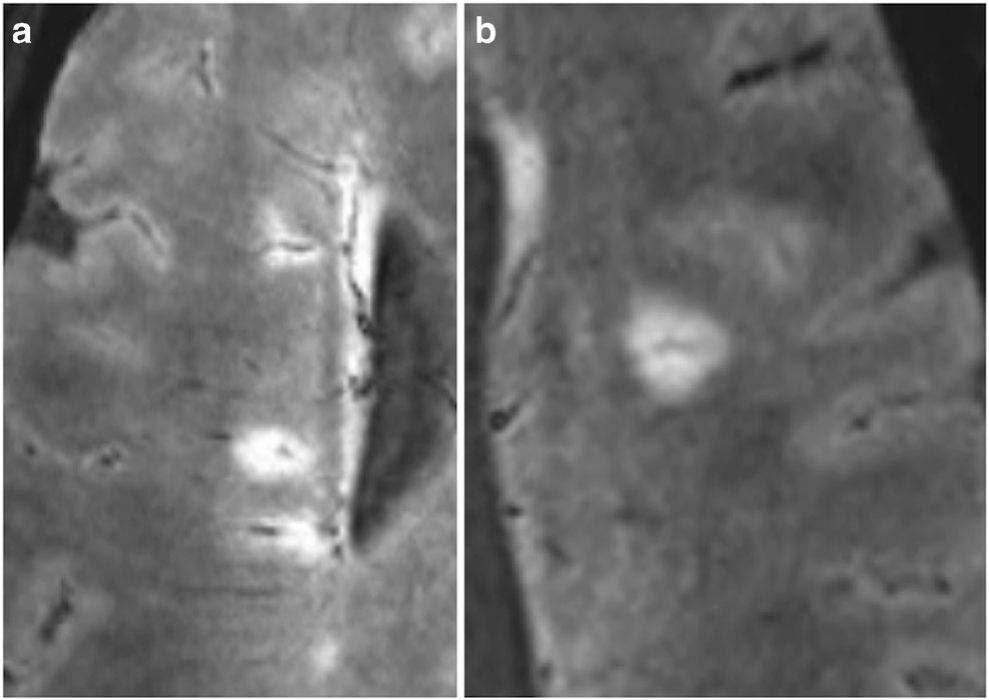
VIL^+^ lesions on FLAIR* in a patient with multiple sclerosis. (**a**) Multiple periventricular VIL+ lesions. (**b**) Subcortical VIL+ lesion. *VIL* vein in lesion

MRI data were assessed by a neuroradiology fellow (RJPS) and a radiology trainee with specific neuroradiology training (TC), supervised by a senior consultant neuroradiologist (JE). Both assessors were blinded to clinical information. WML were defined as discrete areas of high signal intensity on FLAIR* images with a minimum diameter of 3 mm. Their number, location and whether or not they were VIL^+^ (defined as containing a hypo-intense line or dot on axial FLAIR*) were recorded (Fig. 2). Inter-observer agreement of the presence/absence of VIL^+^ WML was assessed on a lesion level using Cohen’sκcoefficient calculated using Microsoft Excel. Additional statistical analysis was performed using StatPlus.

Only WML identified by both observers were included in the analysis. Where there was disagreement between observers about the presence of VIL, images were jointly re-evaluated and a consensus reached. The proportion of VIL^+^ WML was then determined for each patient. A proportion of 45% or more of VIL^+^ WML was considered diagnostic for MS (the ‘VIL45’ criterion) [15]. The proportion in the MS and SVD cohorts was compared using the Mann Whitney test. We also assessed the value of a higher threshold (a proportion of 60% or more; ‘VIL60’ criterion) to determine whether this would impact on sensitivity and specificity.

Recent evidence suggests that a thorough assessment of only a limited number of ‘morphologically characteristic lesions’ (MCLs, i.e. VIL^+^ WML) can be used [15] as an alternative to calculating the relative proportion of VIL^+^ WML. We applied a slightly simplified version of these criteria (‘rule of six criteria’) to our dataset as follows:

1. If there were six or more VIL^+^ WML, a diagnosis of MS was assigned.
2. If there were fewer than six VIL^+^ WML, but VIL^+^ WML outnumbered VIL^−^ WML, a diagnosis of MS was assigned.
3. If neither of these conditions was met, MRI was deemed not confirmatory for a diagnosis of MS.

Finally, datasets of each participant were inspected to determine whether they fulfill the standard DIS and DIT criteria. DIS was considered fulfilled if lesions were identified on T_2_-weighted scans in two or more areas characteristic for MS. DIT was considered fulfilled if both enhancing and non-enhancing WML were present in parallel on the 3D T_1_ post-gadolinium sequence [1].

Sensitivity, specificity and accuracy for the diagnosis of MS were calculated for three VIL-based criteria (VIL45, VIL60 and ‘rule of six’) and two McDonald-based criteria (DIS alone, and DIS and DIT). The McNemar test was used to compare the number of pwRMS and pwSVD who were VIL45^+^ and VIL60 ^+^ with the number of pwRMS who fulfilled the DIS, and DIS and DIT (McDonald 2010 MRI) criteria.

## Results

Datasets of 25 pwRMS (14 men and 11 women; age 41 ± 11 years; disease duration 6 ± 5 years) and 10 pwSVDs (four men and six women; age 59 ± 9 years) were used. pwSVDs were older (p<0.01). Fourteen of 25 pwRMS were on various DMT at the time of scanning.

The inter-observer agreement for determining whether a given WML was VIL^+^, calculated on a lesion by lesion basis, was moderate (κ = 0.70) [19].

In pwRMS, a total of 338 WML were identified (range 5–31; mean 14 ± 7). Of these, 291 WML (86%) were VIL^+^. At least 58% VIL^+^ WML were detected in each pwRMS (range 58–100%; mean 88 ± 12%). All pwRMS met the ‘rule of six’ criteria for MS.

Twenty-four pwRMS (96%) fulfilled the McDonald DIS criterion, whilst three (12%) fulfilled both DIS and DIT.

There was a significant difference in the mean proportion of VIL^+^ WML between pwRMS on DMT and patients who were not (on DMT 83%, not on DMT 94%, p = 0.013).

**Fig. 3:**
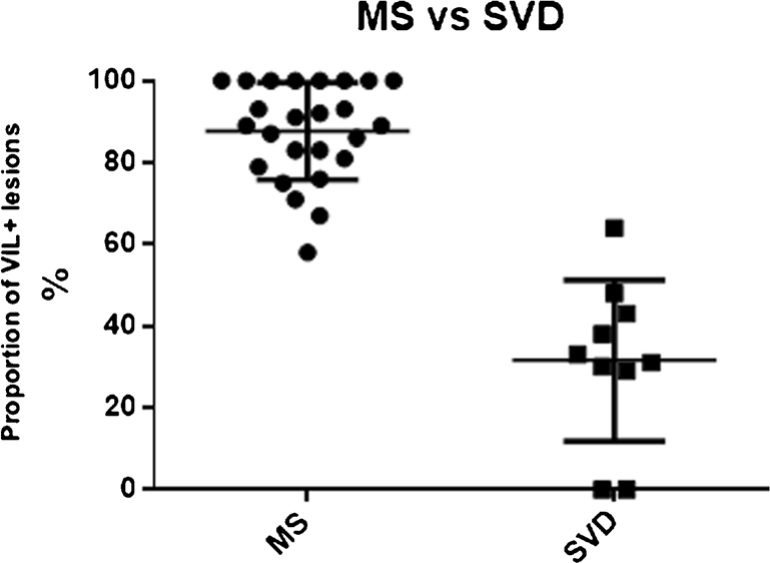
Proportion of VIL^+^ lesions in multiple sclerosis (MS) and cerebral small vessel disease (SVD) cohorts. *VIL* vein in lesion

In pwSVD, a total of 136 WML was identified (range 0–33; mean 13 ± 9). Of these, 54 (40%) were VIL^+^. The mean proportion of VIL^+^ WML was significantly lower (range 0–64%; mean 32 ± 20%) than in pwRMS (p<0.0001) (Fig. 3). Three of ten pwSVDs met the ‘rule of six’ criteria for MS. Six of ten pwSVDs fulfilled the McDonald 2010 DIS criteria; none fulfilled both DIS and DIT.

The VIL45 criterion had a diagnostic sensitivity of 100% and a specificity of 80%. The respective figures for VIL60 were 96% and 90%. The ‘rule of six’ criterion had a sensitivity of 100% and a specificity of 70%.

The McDonald 2010 DIS criterion had a sensitivity of 96% and a specificity of 40% (Table 1).

**Table 1:** Comparison of different criteria for diagnosis of multiple sclerosis (MS)

				Sensitivity (%)	Specificity (%)	Accuracy (%)
				(95% CI)	(95% CI)	(95% CI)
McDonalds DIS Criterion				96	40	80
	Positive	Negative	Total	(80––100)	(12–74)	(63–92)
MS	24	1	25			
SVD	6	4	10			
Total	31	4				
VIL+ 45%				100	80	94
	Positive	Negative	Total	(86–100)	(44–97)	(81–99)
MS	25	0	25			
SVD	2	8	10			
Total	27	8				
VIL+ 60%				96	90	94
	Positive	Negative	Total	(80–100)	(56–100)	(81–99)
MS	24	1	25			
SVD	1	9	10			
Total	25	10				
Rule of Six				100	70	91
	Positive	Negative	Total	(86–100)	(35–93)	(77–98)
MS	25	0	25			
SVD	3	7	10			
Total	28	7				

*CI* confidence interval, *SVD* small vessel disease, *DIS* dissemination in space, *s* vein in lesion

## Discussion

The McDonald MRI criteria are based on a DIS component, which depends on the morphology and distribution pattern of WML, and a DIT component inferred from either (i) the presence in parallel of Gadolinium-enhancing (Gd^**+**^) and non-enhancing (Gd^**−**^) WML at baseline, or (ii) new WML, be they Gd^**+**^ or Gd^**−**^, on follow-up MRI. Although the most recent edition of these criteria has improved and simplified the interpretation of MRI scans to support a diagnosis of MS [1], their applicability in clinical practice remains imperfect [20, 21].

As an alternative to, or perhaps to complement, the pattern-based approach used in the McDonald MRI criteria, the detection and interpretation of VIL^+^ WML *in vivo* benefits from the routine availability of MRI techniques susceptible to deoxygenated blood [22], which appears to enable the visualisation of a histological hallmark of MS WML described for over 160 years, the perivenular morphology of WML [23]. Although we are not aware of any correlative *post mortem* MRI/pathology studies, the topography of hypo-intensity within WML and the presence of deoxyhaemoglobin within veins (which increase susceptibility effects) suggest VIL^+^ WML most likely represent lesions that have emerged around veins.

Using manufacturer-provided sequences acquired at a routine clinical field strength, FLAIR* detected VIL in over 60% of WML in all but one pwRMS included in this study. At a single time point, and using either the threshold of 45% as proposed in earlier studies using 7T [12] and 3T [15] MRI, or a threshold of 60%, the presence of VIL^+^ WML corroborated the diagnosis of MS in all (bar one in the VIL60 analysis) participants studied, as did the ‘rule of six, though with a lower specificity.

The McDonald DIS criterion was as sensitive as the VIL-based indices employed; however, its specificity was significantly lower (40% vs. 70–90%). Whilst 40% appears particularly low, it has previously been shown that DIT information is a key contributor towards specificity in the McDonald MRI criteria [24]. Our study supports previous work in demonstrating a significantly higher proportion of VIL^+^ WML in pwMS compared to pwSVD [12].

However, given that two pwSVDs were VIL45^+^ and one VIL60^+^, the thresholds used were not absolute discriminators. The reason for this may be that veins can incidentally cross SVD lesions thereby giving the wrong impression of a ‘classic’ VIL^+^ WML. Moreover, the lack of post mortem evidence leaves some uncertainty about what exact proportion of hypo-intensities in WML indeed represent veins. On the other hand, VIL^+^ WML in pwMS may be missed due to their small size/diameter. To maximize diagnostic value it may therefore be necessary to combine criteria largely based on lesion morphology with criteria based on distribution pattern and location. A more stringent definition of what constitutes an MS related VIL may also likely be required [10].

**Fig. 4:**
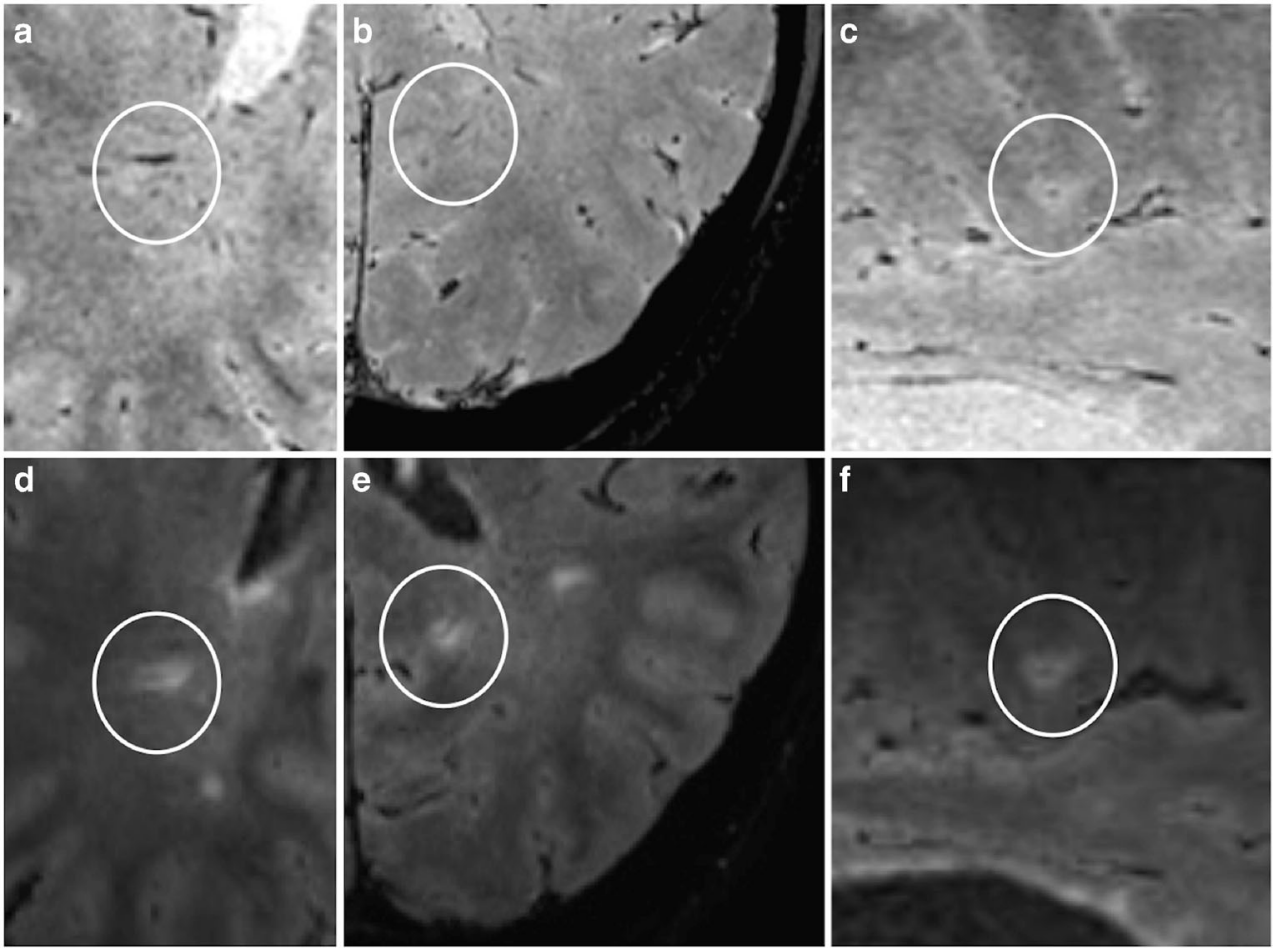
T_2_* versus FLAIR*. (**a–c**) Partial T_2_* slices from three patients with multiple sclerosis (MS): (**a**) axial, (**b**) axial and (**c**) coronal. White circle indicates vein, although lesions not clearly identified. (**d–f**) Corresponding partial FLAIR* slices at same level. White circle indicates white matter lesion around the same vein

The difference in the mean proportion of VIL^+^ WML supports the growing body of evidence suggesting this radiological sign is a useful additional discriminator, in contrast to previous research demonstrating no added benefit [25]. Susceptibility weighted imaging to demonstrate VIL^+^ WML also suggested high sensitivity and specificity for the diagnosis of MS [26, 27]. However, inspection of the FLAIR* maps acquired in our and previous studies [16, 28] suggests there are advantages in combining high isotropic resolution T_2_*-weighted MRI with a well-established technique for WML detection: recognizing VIL^+^ WML on FLAIR* appears more straightforward than on T_2_* alone (Fig. 4). This is in line with another recent study demonstrating that using FLAIR and FLAIR* as part of a global assessment of whether >40% WML contain a vein improves diagnostic accuracy for MS without the need to assess every single lesion [28].

### Limitations

Though image reviewers were often unaware of the diagnosis, systematic blinding was not undertaken. Moreover, most pwRMS were on DMT when MRI datasets were acquired, compromising the potential to reveal Gd^+^ WML and likely explaining the particularly small number (3) of pwRMS in our study meeting DIT criteria at a single time point [24]. A limitation inherent in the method is that identification of VIL^+^ WML remains to some extent subjective; although agreement in our study between raters was moderate (κ = 0.70), specific training and reading criteria may be required to introduce the VIL sign as a standard in clinical practice.

The acquisition protocol of our study was slightly different from previous studies using similar methodology [16, 28]. In particular, manual administration of the contrast medium followed by the T_2_* sequence, and a different dosing regime (standard instead of weight-adjusted dose) may have altered the vein versus lesion contrast with a possible impact on the VIL45 and VIL60 indices and inter-rater agreement.

Finally, pwSVD were older than those in the MS group, thus providing an imperfect match for the pwRMS cohort. However, higher numbers of WML are to be expected with age, and as such older people should represent a more challenging comparator group to test the VIL criteria. Future studies should compare FLAIR* with McDonald criteria in a larger cohort of people where MS is the suspected diagnosis, i.e. people with a first manifestation of symptoms and/or signs suggestive of demyelination, and acquire FLAIR* at the time of presentation and after defined subsequent intervals. FLAIR* has already shown promise in differentiating between MS and migraine, for example [29].

## Conclusion

In a clinical setting, and using standard manufacturer-supplied sequences, FLAIR* at 3T reliably enabled *in vivo* detection of VIL^+^ WML, which resemble a characteristic histological feature of MS. All pwRMS were VIL45^+^, underpinning previous data acquired in a research setting. The mean proportion of VIL^+^ WML was significantly higher in pwRMS compared to pwSVD.

Our data suggest that using either VIL45 or VIL60 is as sensitive, and potentially more specific, for the diagnosis of MS than current McDonald 2010 MRI criteria. FLAIR* may thus support the development of more accurate and easy to use MRI criteria for a diagnosis of MS. In line with the recent first consensus statement on the evaluation of central veins in WML [10], further prospective and comparative studies are required to confirm the diagnostic value of VIL, and the techniques used to visualize these and other features of MS.

## References

[1] Polman CH, Reingold SC, Banwell B (2011). Diagnostic criteria for multiple sclerosis: 2010 revisions to the McDonald criteria. Ann Neurol.

[2] Charil A, Yousry TA, Rovaris M (2006). MRI and the diagnosis of multiple sclerosis: expanding the concept of “no better explanation”. Lancet Neurol.

[3] Kappos L, Freedman MS, Polman CH (2007). Effect of early versus delayed interferon beta-1b treatment on disability after a first clinical event suggestive of multiple sclerosis: a 3-year follow-up analysis of the BENEFIT study. Lancet.

[4] Coles AJ, Cox A, Le Page E (2006). The window of therapeutic opportunity in multiple sclerosis: evidence from monocolonal antibody therapy. J Neurol.

[5] Leist TP, Comi G, Cree BA (2014). Effect of oral cladribine on time to conversion to clinically definite multiple sclerosis in patients with a first demyelinating event (ORACLE MS): a phase 3 randomised trial. Lancet Neurol.

[6] Geraldes R, Esiri MM, Deluca GC et al. (2016) Age-related small vessel disease: a potential contributor to neurodegeneration in multiple sclerosis. Brain Pathology 10.1111/bpa.12460 PMC8029132 27864848

[7] Giovannoni G, Turner B, Gnanapavan S, Offiah C, Schmierer K, Marta M (2015). Is it time to target no evident disease activity (NEDA) in multiple sclerosis?. Multiple Sclerosis Relat Disord.

[8] Natalizumab for the treatment of adults with highly active relapsing-remitting multiple sclerosis. NICE technology appraisal guideline [TA127]. Available at: http://www.nice.org.uk/guidance/TA127/chapter/1-guidance. Accessed on: June 2015

[9] Lassmann H (2005). Multiple sclerosis pathology: evolution of pathogenetic concepts. Brain Pathol.

[10] Sati P, Jiwon O, Todd Constable R, et al. (2016) The central vein sign and its clinical evaluation for the diagnosis of multiple sclerosis: a consensus statement from the North American Imaging in Multiple Sclerosis Collective. Nat Rev Neurol 10.1038/nrneurol.2016.166 27834394

[11] Tan IL, van Schijndel RA, Pouwels PJ (2000). MR venography of multiple sclerosis. AJNR.

[12] Mistry N, Dixon J, Tallantyre E (2013). Central veins in brain lesions visualized with high-field magnetic resonance imaging. JAMA.

[13] Tallantyre EC, Dixon JE, Donaldson I (2011). Ultra-high-field imaging distinguishes MS lesions from asymptomatic white matter lesions. Neurology.

[14] Tallantyre EC, Morgan PS, Dixon JE (2009). A comparison of 3T and 7T in the detection of small parenchymal veins within MS lesions. Invest Radiol.

[15] Mistry N, Abdel-Fahim R, Samaraweera A (2015). Imaging central veins in brain lesions with 3-T T2*-weighted magnetic resonance imaging differentiates multiple sclerosis from microangiopathic brain lesions. Mult Scler.

[16] Sati P, George IC, Shea CD, Gaitan MI, Reich DS (2012). FLAIR*: a combined MR contrast technique for visualizing white matter lesions and parenchymal veins. Radiology.

[17] Staals J, Makin SDJ, Doubal FN (2014). Stroke subtype, vascular risk factors, and total MRI brain small-vessel disease burden. Neurology.

[18] Sati P, Thomasson DM, Lin N (2014). Rapid, high-resolution, whole-brain, susceptibility-based MRI of multiple sclerosis. Mult Scler.

[19] McHugh ML (2012). Interrater reliability: the kappa statistic. Biochem Med (Zagreb).

[20] Hawkes CH, Giovannoni G (2010). The McDonald criteria for multiple sclerosis: time for clarification. Mult Scler.

[21] Schmierer K, Turner BP (2012). The second revision of ‘McDonald’ criteria for the diagnosis of multiple sclerosis. ACNR.

[22] Mittal S, Wu Z, Neelavalli J, Haacke EM (2009). Susceptibility-weighted imaging: technical aspects and clinical applications, part 2. AJNR.

[23] Charcot J-M (1868). Histologie de la sclérose en plaques. La Lancette Française, Gazette des Hôpitaux Civils et Militaires.

[24] Swanton JK, Rovira A, Tintore M (2007). MRI criteria for multiple sclerosis in patients presenting with clinically isolated syndromes: a multicentre retrospective study. Lancet Neurol.

[25] Lummel N, Boeckh-Behrens T, Schoepf V, Burke M, Bruckmann H, Lin J (2011). Presence of a central vein within white matter lesions on susceptibility weighted imaging: a specific finding for multiple sclerosis?. Neuroradiology.

[26] Lane JI, Bolster B, Campeau NG, Welker KM, Gilbertson JR (2015). Characterization of multiple sclerosis plaques using susceptibility-weighted imaging at 1.5T: can perivenular localization improve specificity of imaging criteria?. J Comput Assist Tomogr.

[27] Kilsdonk ID, Wattjes MP, Lopez-Soriano A (2014). Improved differentiation between MS and vascular brain lesions using FLAIR* at 7 Tesla. Eur Radiol.

[28] George IC, Sati P, Absinta M (2016). Clinial 3-tesla FLAIR* MRI improves diagnostic accuracy in multiple sclerosis. Mult Scler.

[29] Solomon AJ, Schindler MK, Howard DB (2015). “Central vessel sign” on 3T FLAIR* MRI for the differentiation of multiple sclerosis from migraine. Ann Clin Transl Neurol.

